# Defining health data elements under the HL7 development framework for metadata management

**DOI:** 10.1186/s13326-022-00265-5

**Published:** 2022-03-18

**Authors:** Zhe Yang, Kun Jiang, Miaomiao Lou, Yang Gong, Lili Zhang, Jing Liu, Xinyu Bao, Danhong Liu, Peng Yang

**Affiliations:** 1 Institute for Health Informatics, Department of Health Statistics, the Ministry of Education Key Lab of Hazard Assessment and Control in Special Operational Environment, School of Public Health, Fourth Military Medical University, 169 Changle West Road, Xi’an, 710032 China; 2 Information Center, First Affiliated Hospitals, Fourth Military Medical University, 15 Changle West Road, Xi’an, 710032 China; 3 School of Biomedical Informatics, The University of Texas Health Science Center, 7000 Fannin, Houston, TX 77030 USA; 4 Center for Health Statistics and Information, National Health Commission of the People’s Republic of China, No. 1, Xizhimenwai South Road, Xicheng District, Beijing, 100044 China; 5 Network Management Office, Armed Police Shaanxi General Corps Hospital, 88 South Second Ring Eastern Section, Xi’an, 710054 China; 6 Information Technology and Service Business, Sinosoft Company Limited, 6 Zhongguancun South Street, Haidian District, Beijing, 100080 China

**Keywords:** Data element, Metadata, Standards, Health level seven, RIM

## Abstract

**Background:**

Health data from different specialties or domains generallly have diverse formats and meanings, which can cause semantic communication barriers when these data are exchanged among heterogeneous systems. As such, this study is intended to develop a national health concept data model (HCDM) and develop a corresponding system to facilitate healthcare data standardization and centralized metadata management.

**Methods:**

Based on 55 data sets (4640 data items) from 7 health business domains in China, a bottom-up approach was employed to build the structure and metadata for HCDM by referencing HL7 RIM. According to ISO/IEC 11179, a top-down approach was used to develop and standardize the data elements.

**Results:**

HCDM adopted three-level architecture of class, attribute and data type, and consisted of 6 classes and 15 sub-classes. Each class had a set of descriptive attributes and every attribute was assigned a data type. 100 initial data elements (DEs) were extracted from HCDM and 144 general DEs were derived from corresponding initial DEs. Domain DEs were transformed by specializing general DEs using 12 controlled vocabularies which developed from HL7 vocabularies and actual health demands. A model-based system was successfully established to evaluate and manage the NHDD.

**Conclusions:**

HCDM provided a unified metadata reference for multi-source data standardization and management. This approach of defining health data elements was a feasible solution in healthcare information standardization to enable healthcare interoperability in China.

## Background

Accurate and comprehensive information structures are the key point for biomedical and healthcare information exchanges. To realize information sharing, there must be a standardized method to represent the information. Novel patterns developed for this representation makes semantic information sharing a reality. The ontology is the most popular method that provides the basis for the information model classes [1, 2]. Information models that express the relationships among classes can provide an accurate context for data semantics expression [3, 4]. The Health Level Seven International (HL7) standards have become universal for the exchange, integration, sharing and retrieval of health information [5–7]. The HL7 Development Framework (HDF) is a framework for modelling and administrative processes, and deliverables used by HL7 to produce specifications that are used by the healthcare information management community to overcome challenges and barriers to interoperability among computerized healthcare-related information systems [8–11]. HL7 version 3 (v3) is based on HDF methodology and generates messages and electronic documents for the clinical information exchange [12–15]. The HL7 Reference Information Model (RIM) which is the main core in HL7 v3 covers all aspects of healthcare information and can be compatible with existing data standards and knowledge models and thus can serve as the foundation for information integration across platforms and systems [9, 16, 17]. RIM defines a series of classes and subclasses, attributes, data types and value domains related to medical activities; furthermore, RIM provides a clear, common context and semantics that all standards and norms can cohere with [6, 18]. RIM has been introduced to China and released as a national standard in 2013 [19]. There have been ongoing efforts in RIM modelling and application, most of which focus on ontological engineering of RIM [20–22], clinical data interoperability [23–26], domain knowledge representation [27–30], database development [31], and knowledge and data integration [29, 32], while few studies seek to implement and validate RIM for data collection and management on the countrywide level.

Chinese Health Standards Commission developed and issued a health data element dictionary in 2011 as a national health data standard [33]. The dictionary gathers data elements (DEs) recorded and collected in various domains of health sectors. DEs were described through six properties, including data element identifier, name, definition, permitted values, data type and format [34, 35]. However, some DEs are mutually inclusive, intersect, or overlap because they usually come from different business collection forms (e.g. chronic disease management, planned immunization, women’s healthcare). The consistency and comparability for data exchange and sharing cannot be guaranteed [36]. Moreover, with further development of health services demands and information technologies, more DEs will be created from different fields, projects and organizations. The infinite increase of DEs poses a challenge for their centralized management and standardization.

Healthcare data management is a domain with various proposed solutions and knowledge that accumulated through years of research. Many efforts which try to facilitate information semantic interoperability have already been developed. HL7 Fast Health Interoperability Resources (FHIR) takes a modular approach and represents the atomic/ granular healthcare data (e.g., heart rate, procedure, medication, allergies) as independent modular entities. The main advantage of FHIR is that it’s easier to implement as it uses an API-based approach and a choice of JSON or XML or RDF for representing the data [37, 38]. The IHE Data Exchange (DEX) profile proposed a metadata registry to search and retrieve metadata definitions, and flexible mapping between clinical research and patient care data elements [39]. The ISO/IEC 11179 model provides a standard metadata model for the representation of data elements and provides a methodology for the registration of the descriptions of data elements through this standard model to the metadata registries [40].

Although these standards have a good foundation in enabling semantic interoperability for healthcare data, we continue to use the methodology of HL7 v3 when building the NHDD for three main reasons: firstly, HL7 v3 adopts a series of information models and graphical modeling methods to ensure standard coding and implementation, and enabling semantic interoperability through defined terms and data types. Secondly, RIM is the core of HL7 v3 and highly abstract. It is an international shared information model and is also the root of all information models and structures in v3 development process. Lastly, most importantly, HL7 RIM has been adopted in China and already become a national standard, and is now widely used in the construction of many Chinese medical information systems. To avoid large changes and maintain the consistency of the existing series of standards and applications in China, we continue to use the methodology of HL7 v3 and customize the metadata.

In view of international experiences and general applicability of HL7 methodology in healthcare fields, this study is intended to develop a Health Concept Data Model (HCDM) and National Health Data Dictionary (NHDD) based on HL7 RIM and HDF methodology, and then to develop a model-based information system for convenient metadata collection and management, with the aim to facilitate healthcare information standardization and healthcare interoperability in China.

## Implementation and result

### HCDM structure and definition

The HCDM adopted three-level architecture of HL7 RIM: *class*, *attribute* and *data type*. *Class* describes aspects of the health and care business with their significant characteristics through their *Attributes* and their relationships to other *Classes*. *Attribute* describes the properties of *Classes* and provide common data definitions for classes. *Data type* defines the allowable values of attributes and what these values “mean”.

### HCDM metadata and comparison with HL7 RIM

The construction of HCDM mainly came from HL7 RIM and was adapted based on the needs of the national health system (Table 1). Firstly, six classes and their attributes directly used contents of HL7 RIM. Then 4640 data items from 55 data sets of national health system were classified (through Chinese text classification toolkit THUCTC launched by the Natural Language Processing Laboratory of Tsinghua University [41, 42]) into these six classes of HCDM. Lastly, sub-classes and attributes of HL7 classes were adjusted and optimized according to actual classification results.

**Table 1:** 55 data sets and 7 health business domains

Domain	Data set	Domain	Data set
**Electronic Medical Record**	01: medical record summary 02: outpatient and emergency medical record 03: outpatient and emergency prescription 04: examination and laboratory test record 05: general therapy and treatment record 06: delivery record of therapy and treatment 07: nursing operation records 08: nursing evaluation and plan 09: informed consent 10: first page of inpatient medical record 11: first page of inpatient medical record summary of traditional Chinese medicine 12: admission record 13: inpatient progress note 14: inpatient order 15: discharge summary 16: transfer record	**Disease Control**	01: HIV/AIDS prevention and control 02: schistosomiasis management 03: chronic filariasis management 04: occupational disease report 05: occupational health surveillance 06: behavioral risk monitoring 07: medical certificate of death 08: infectious disease report 09: tuberculosis report 10: immunization 11: tuberculosis (TB) management 12: tuberculosis (TB) patient drug-resistant management 13: adverse event following immunization 14: vaccine management 15: registration and report of stroke 16: management of stroke patient 17: cervical cancer screening registry 18: colorectal cancer screening registry
		**Medical Service**	01: outpatient summary 02: hospitalization patient summary 03: adults health examination
**Disease** **Management**	01: hepatitis B patients management 02: hypertension patients management 03: severe psychiatric disease patients management 04: elderly health management 05: type 2 diabetes patients health management 06: cancer patients management	**Children’s Health**	01: birth certificate 02: children’s physical examination 03: new born screening 04: nutritional disease of children management
		**Women’s Health**	01: premarital care 02: screening common gynecological diseases 03: technical service of family planning 04: maternal healthcare and high-risk management 05: prenatal screening and diagnosis 06: birth defect monitoring
**Basic Health** **Record**	01: health record for residents 02: residents’ health card		

### Class

HCDM has the same backbone with six major classes of HL7 RIM: *Entity, Role, Rolelink, Participation, Act, Act Relationship*. In HCDM, *Entity* represents the physical things and beings that are of interest to, and take part in health care*. Role* establishes the roles that entities play as they participate in health care acts*. Rolelink* represents relationships between individual roles*. Participation* expresses the context for an act in terms such as who performed it, for whom it was done, where it was done, etc. *Act* represents the actions that are executed and must be documented as health care is managed and provided*. Act Relationship* represents the binding of one act to another, such as the relationship between an order for an observation and the observation event as it occurs.

Based on classification results, HCDM reduced 11 subclasses (*Entity-living subject, Role-patient, Role-LicensedEntity*, *Role-Access*, *Participation-ManagedParticipation, Act-Observation-diagnosticImage, Act-Supply-Diet, Act-Account, Act-ControlAct, Act-Device Task* and *Act-Working list)* and added one subclass (*Act-Exposure*) to RIM because currently no data is essentially attributed to those reduced subclasses (e.g., *Act-ControlAct*, *Act-Device Task*, *Role-patient*, *Role-LicensedEntity*). The added subclass (*Act-Exposure*) which is not listed separately in RIM is currently indispensable for health data management. Classes *RoleLink* and *ActRelationship* have no subclasses in HCDM and RIM. Finally, HCDM has 14 subclasses/secondary classes and 1 tertiary class, while RIM has 21 subclasses and 5 tertiary classes (Table 2).

**Table 2:** Class comparison and reasons for differences between HCDM and HL7 RIM

HCDM	HL7 RIM	Reasons for Differences
**Entity**	**Entity**	*Person* is a subclass of *Living Subject* in RIM. Considering the applicability of health metadata management, we moved up one level and directly adopted *Person* as the subclass of Entity.
Organization	Organization	
	Living Subject	
Person	Person	
	NonPersonLivingSubject	
Place	Place	
Material	Material	
**Role**	**Role**	Contents of subclasses *Patient*, *LicensedEntity*, and *Access* can be expressed through vocabularies *Role class code* and *role code* in HCDM.
	Patient	
Employee	Employee	
	LicensedEntity	
	Access	
**RoleLink**	**RoleLink**	
**Participation**	**Participation**	*ManagedParticipation* is the subclass of *Participation* to constrain the participation status, which is not concerned in HCDM.
	ManagedParticipation	
**Act**	**Act**	*ControlAct* in RIM is used to regulate the content of the transaction contract, and it is no corresponding data in HCDM. Also, no data is currently attributed to *Device Task*, *Working List*, *diet* and *Account* in HCDM. The HCDM includes 24 disease control and management datasets, in which risk factor exposure is the indispensable information, so a special class *Exposure* is added.
Observation	Observation	
PublicHealthCase	PublicHealthCase	
	DiagnosticImage	
Procedure	Procedure	
Substance Administration	Substance Administration	
Patient Encounter	Patient Encounter	
Supply	Supply	
	Diet	
	Account	
Invoice Element	InvoiceElement	
FinancialTransaction	FinancialTransaction	
FinancialContract	FinancialContract	
	ControlAct	
	Device Task	
	Working List	
Exposure		
**ActRelationship**	**ActRelationship**	

### Attribute

Attributes of classes in HL7 RIM were also adjusted and trimmed according to the data classifications. Some attributes of classes and subclasses were added or removed in HCDM. For example, *administrative division code* (used for identifying national administrative districts) and *housing type code* (used for differentiating family housing types) were added attributes, and *RiskCode* in class “Entity” was removed because there are no entities about risk information in collected data sets. Eventually, compared with HL7 RIM, 8 attributes which meet current needs of different health fields were added in HCDM including *person-nationality code*, *person-household type code*, *organization-administrative division code*, *organization-level code*, *organization-type code*, *employee-family income per capita*, *financial transaction-payer code*, *financial transaction-way of payment code*. The comparison of attributes of class “Entity” between HCDM and RIM are shown in Table 3.

**Table 3:** Attributes of class *Entity* between HCDM and HL7 RIM

HCDM	HL7 RIM
**Entity**	**Entity**
classCode	classCode
determinerCode	determinerCode
id	id
code	code
quantity	quantity
name	name
desc	desc
existenceTime	existenceTime
telecom	telecom
–	StatusCode
–	RiskCode
–	handlingCode
**Entity—organization**	**Entity—organization**
typeCode	–
levelCode	–
addr	addr
administrativeDivisionCode	–
–	standardIndustryClassCode
**Entity—Person**	**Entity- Living Subject-Person**
addr	addr
maritalStatus	maritalStatus
educationLevelCode	educationLevelCode
genderCode	genderStatusCode
birthTime	birthTime
nationalityCode	–
ethnicGroupCode	ehtnicGroupCode
religiousAffiliationCode	religiousAffiliationCode
householdTypeCode	–
deceasedTime	deceasedInd
autopsyInd	organDonorInd
–	disabilityCode
–	livingArrangementCode
–	raceCode
**Entity—Place**	**Entity—Place**
moblieInd	moblieInd
addr	addr
directionsText	directionsText
positionText	positionText
**Entity—Material**	**Entity—Material**
formCode	formCode

### Data type

Metadata’s data types were referenced to Data Types Specification (R2) [43] of HL7 RIM and made some adjustments. The HL7 v3 data type is purely semantic and the hierarchical structure and attributes’ data types are in the relative high level. In HCDM, the abstract principle is using lower (more specific) rather higher (more general) level at the same condition in order to facilitate formal expression of DEs. Eventually, there are 16 data types in HCDM as follows: *II*, *ED, BL, INT, PQ, Real, MO, URLST, TS, AD, EN, CS, CV, CE, CD* and *ANY*. Some data types are so fundamental that there are no distinguishable semantic components (e.g. BL). The composite data types contain additional data types that are referenced as components or subcomponents (e.g. PQ:value and unit). The attribute *ANY* is usually avoided to use if possible for its unspecific attribute expression. The data type of the same attribute is also different between in HCDM and in HL7 RIM.

**Fig. 1:**
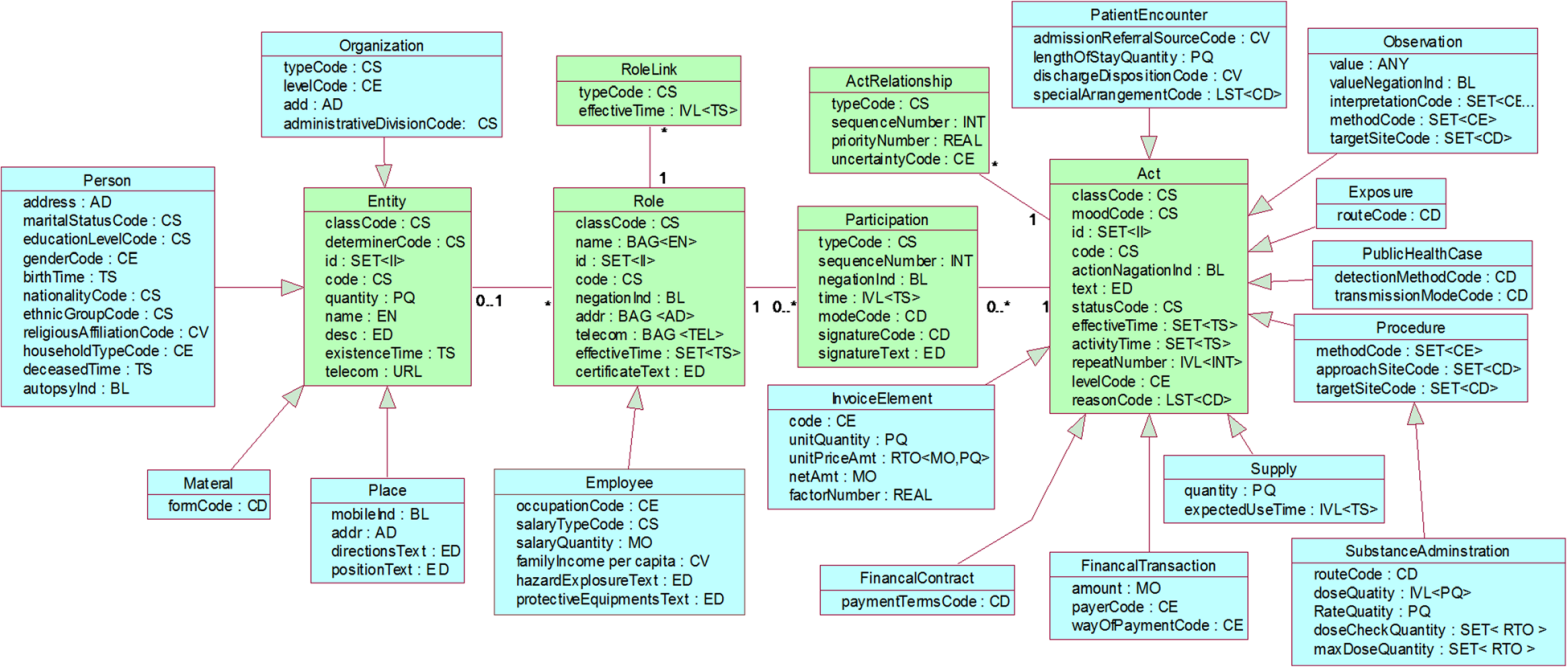
Framework of HCDM. HCDM has 6 classes, 15 sub-classes,100 attributes and 100 data types. Each class has several attributes and data types to represent its semantics. The green rectangles represent parent classes and the blue ones represent sub-classes. Hollow arrows represent the inheritance relationship from parent class to child class

In total, HCDM was developed with 6 classes, 15 sub-classes, 100 attributes and 100 data types. Its framework was expressed by the Unified Modelling Language and shown in Fig. 1, which has been issued as a China’s health industry standard in May 2020 [44].

## Data elements derived from HCDM and their description

Data elements were derived by constraining metadata (*Class*, *Attribute* and *Data type*) in HCDM and described according to ISO/IEC 11179 metamodel which defines how a data element can be classified and semantically described, named, identified, stored, retrieved, and managed [45, 46]. A data element comprises two parts in ISO/IEC 11179 metamodel: Data Element Concept and Value Domain. A Data Element Concept joins an Object class (like a person) with its Property (like sex) [47]. The Value Domain is the set of permissible values for one or more data elements. The mapping concept of ISO/IEC 11179 metamodel to HCDM are as follows: the *Object Class* in ISO/IEC 11179 metamodel corresponds to the Class in the HCDM, the *Property* of Object Class corresponds to the Attribute of Class, and the *data type of Value Domain* corresponds to the Data Type of attribute in the HCDM (Table 4).

**Table 4:** Mapping relationship between ISO/IEC 11179 metamodel and HCDM

HCDM	ISO/IEC 11179 metamodel
Class	*DE:Object class*
Attribute	*DE:Property*
Data type	*DE:data type of Value domain (DE:data type)*

Based on the HCDM, national health data dictionary (NHDD), which includes three types of DEs (initial DE, general DE, domain DE), was developed and has also been issued as a China’s health industry standard in May 2020 [48]. Initial DEs were formed by the combination of classes, attributes and data types in HCDM. General DEs were generated by de-composing the semantic components of data types of initial DEs. Domain DEs were defined or specified by constraining general DEs through terms in controlled vocabulary.

### Initial data elements

**Fig. 2:**
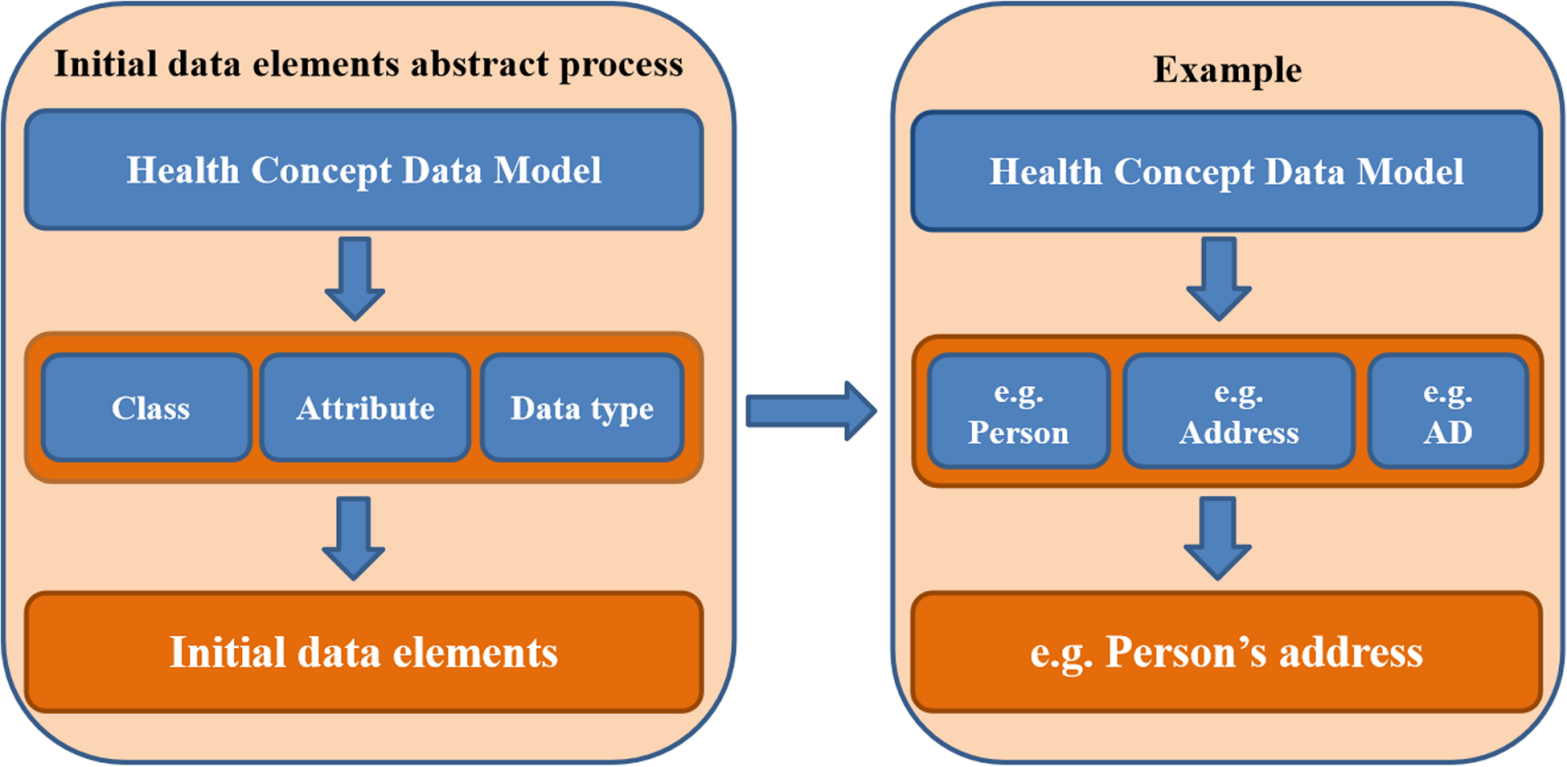
Abstract process of initial data elements. The left side indicates the initial data elements abstract process, and the right side shows an example for initial data element *person’s address*, which is formed by constraining the object class “person”, the attribute “address” of person and data type “AD” in the Health Concept Data Model

100 initial DEs were extracted from HCDM and represented through data types (foundation, basic and quantities). The initial DEs serves as a bridge between the HCDM and general DEs, and so they have no corresponding specification on the semantic expression. As shown in Fig. 2, the initial DE *person’s address* is formed by constraining the Class (*DE:Object class*) “person”, Attribute (*DE:Property*) “address” of person and the Data type (*DE:data type*)"AD”.

### General data element

General DEs are independent of specific domain context to be maintained at a higher level. 144 general DEs were developed from initial DEs. The mapping method from ISO/IEC 11179 metamodel to the HCDM was as the same as initial DE’s derivation. But data types of general DEs were developed through further specializing initial DEs’data types. Basing on initial DEs’ data types, we unfolded the components of HCDM data types. The general DE was then formed by the combination of initial DE and each unfolded components of Data Type.

Such specialization mainly aimed at ANY which is the data type for value from medical observation. ANY can be specified into quantitative measurements, liter, index values, ranges, ordinals, nominal, etc. Based on actual demand, 19 metadata items were adopted in this work from ISO/IEC 11179 to describe general DEs. Table 5, taking Person Nationality Code as an example, presents standardized description of the general DE.

**Table 5:** Standardized description of general DE *Person Nationality Code*

Metadata	Value
Metadata name	Person Nationality Code
**Data element attributes**	
Metadata type	General Data element
Specification name	Person Nationality Code
Synonyms	nationality code
Metadata identifier	655217
Register status	Draft
Definition	From a legal sense to person’s definition of nation. In general, if a person has the nationality means that the individual is legal citizen in the country.
Data type	CS
Register organization	Centre for Health Statistics and Information of National Health Commission of China
Version	V1.0
Use^a^	(1) Children’s HealthDataSet-01: birth certification; (2) Disease Management Data Set-01: hepatitis B patients management; (3) Women’s Health Data Set-01: premarital health examination
**Data element concept attributes**	
Data element concept^b^	Person’s nationality
Object class^c^	Person
Object class identifier	PersonID
Property^d^	Nationality
Property identifier	NationalityID
**Value domain attribute**	
Format	Code
Classification Schema	GB/T 2659–2000 [49]
Classification Schema Identifier	Person Nationality Code ID

Comments: a:datasets which use this data element. b:concept that can be represented in the form of a data element, described independently of any particular representation (see ISO/IEC 11179–3). c:set of ideas, abstractions, or things in the real world that are identified with explicit boundaries and meaning and whose properties and behavior follow the same rules. d:characteristic of an object or entity.

In addition, six categories of representation format for general DEs were also defined according to ISO/IEC 11179–3: text, symbols, values, date, time and code. When some similar DEs appeared repeatedly, only one DE was retained such as code system identifiers and system names which repeated in all general DEs with coded attribute (entity class code, entity code, role code, act code, etc.), only one code system identifiers and system names was retained in NHDD.

### Domain DE and Controlled vocabulary

General DEs are largely independent of specific domain context and usually need to be localized before being adopted by domain data developers. Such localization should follow a unified rule to avoid semantic confusion for information sharing. Controlled vocabularies were developed on the basis of the standard Health Information Value Codes (standard number: WS 364) and by referring to HL7 vocabularies [50]. There are currently 12 controlled vocabularies in NHDD: *Entity classCode and Entity code, EntitydeterminerCode, Entity URLScheme, Entity telecommunicationAddressUse, Person addressType, Role classCode and Role code, Rolelink code, Participation typeCode, Act classCode and Act code, Act moodCode, Act relationshipCode, and Act statusCode.*

The Entity *classCode* for each object class provides all possible subtypes (can be further subdivided) or instance (can’t be further subdivided) of the object class for localization of the general DEs. The controlled vocabulary *Entity classCode* provides restrictions for general DEs to be specified into one or more domain DEs. Entity is specialized into instances of human, microorganisms animals plants listed in the controlled vocabularies for the general DEs of *Entity classCode* and *Entity Code*. The link between Controlled vocabularies *Entity Class Code* and *Entity Code* is shown in Table 6 in which codes are the permissible value set for *classCode* and *code* of “Entity” in Fig. 1.

**Table 6:** Controlled vocabularies *Entity Class Code* and *Entity Code*

EntityclassCode	Entity code	Concept ID^a^
Organizations		E402924
	Public agencies	E552357
	Administrative areas	E858133
Organism		E631881
	Humans	E545147
	No-human living	E568177
	Microorganisms	E373479
	Animals	E367680
	Plants	E827127
Material		E224432
	Material for manufacture	E799047
	Containers	E570708
	Devices	E692167
	Chemicals	E475018
	Food	E276604
Place		E239241
	Nations	E740660
	Province (Autonomous region, Municipalities)	E777781
	District (City,State,League)	E761880
	Country (District,Banner)	E749454
	Towns (Streets)	E385646
	Villages (Neighbourhood committees, Community)	E577135

Comments: a:The concepts in NHDD are coded an unique concept ID, and can be identified and managed through this ID. The first letter in the concept ID is the first letter of the object class which the concept belongs, and the next six digits number is an unique random number and created by a computer program.

**Fig. 3:**
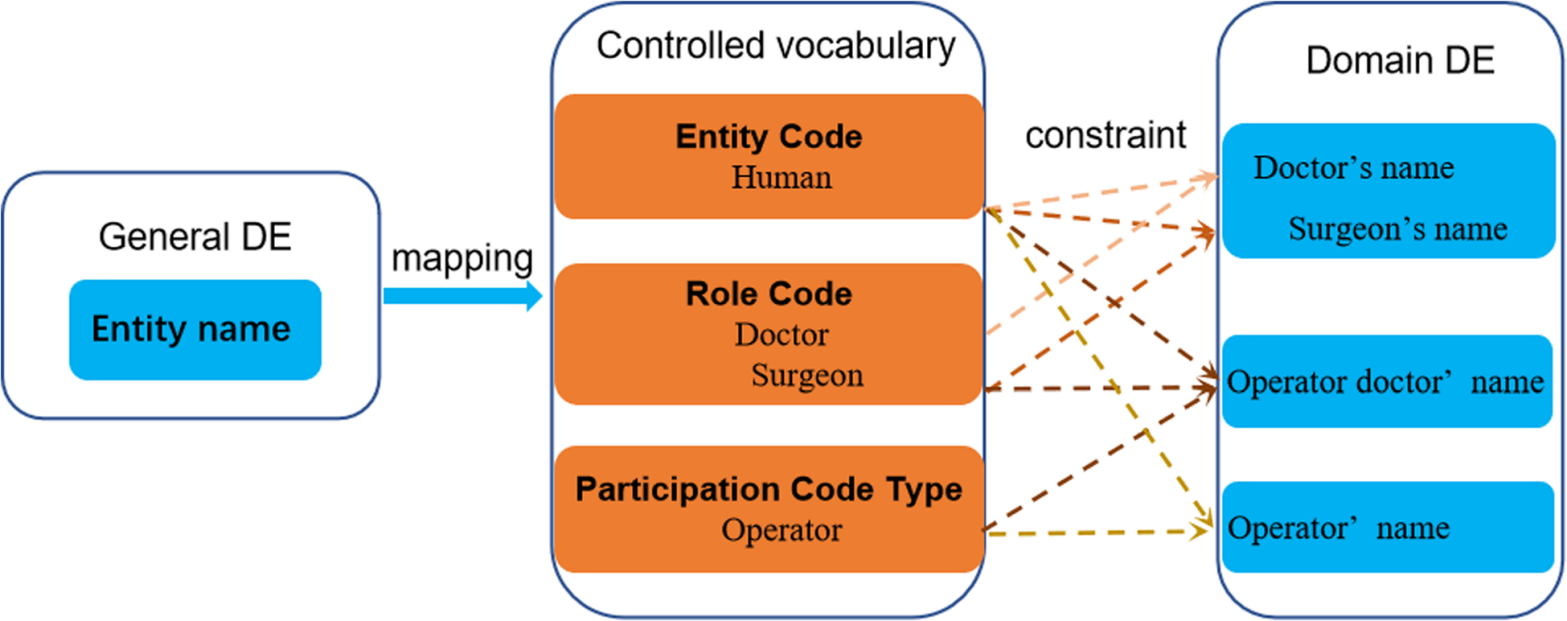
The relationship of general DE, controlled vocabulary and domain DE. “Entity name” of general DE can be constrained to the domain DE “doctor’s name” based on the term “doctor” and the domain DE “surgeon’s name” based on the term “surgeon” (subtype of “doctor”) in the vocabulary of “roleCode”, and to “operator’s name” based on the term “operator” in the vocabulary of “participationCodeType”. The “entity name” ofgeneral DE can also be constrained to the domain DE “operation doctor’s name” based on the vocabularies combination (pre-coordinated) of the “roleCode (term: doctor)” and “participationCodeType (term: operator)”

Consequently, related general DEs can be constrained into specific domain DEs. As shown in Fig. 3, “Entity name” of general DE can be constrained to a domain DE “doctor’s name” based on the term “human”, “doctor”, and to a domain DE “surgeon’s name” based on the term “human”, “surgeon” (subtype of “doctor”) in the vocabulary of “*Entity Code*” and “*Role Code*”, and to “operator’s name” based on the term “human”, “operator” in the vocabulary of “*Entity Code*” and “*Participation Code Type*”. The “Entity name” of general DE can also be constrained to a domain DE “operation doctor’s name” based on the vocabularies combination (pre-coordinated) of the “*Entity Code* (term: human)”, “*Role Code* (term: doctor)” and “*Participation Code Type* (term: operator)”.

In total, domain DEs are standardized through 22 metadata items, including 14 data element attributes and 6 value domain attributes, which are all from the ISO/IEC11179 model. Among them, the metadata item named “Metadata Reference” can be related to NHDD and the “Relation Type” can be constrained to the class in HCDM. Value domain attributes indicate the relationship between domain DEs and controlled vocabularies.

The relationships of HCDM, initial DEs, general DEs and domain DEs are shown in Table 7.

**Table 7:** The process of forming initial data elements, general data elements and domain data elements in the class “Entity”

HCDM		*Initial data element*	HCDM Data type			*General data element*	Terms in Controlled Vocabularies	*Domain data element*
class	attribute		name	component	format			
Entity class	classCode	*Entity Class Code*	CS	Code	Code	*Entity Class Code*	Organizations	*Organizations code* *Public agencies code*
	determinerCode	*Entity DeterminerCode*	CS	Code	Code	*Entity Determiner Code*	Humans	*General described for person*
	id	*EntityII*	Set<II>	root UID/OID	Symbol	*Entity II UID/OID*	Organizations	*ID UID*
				Extension	Symbol	*Entity II*	Organizations	*ID number*
				identifierName	Text	*Entity Identifier Name*	Organizations	*ID name*
	code	*Entity Code*	CE	Code	Code	*Entity Code*	Organizations	*Organization code*
	quantity	*Entity Quantity*	PQ	Value	Values	*Entity Quantity*	Humans Organizations Material	*The number of people* *The number of institutions* *Number of Chinese medicine* *Tablets to drink (agent)*
				Unit	Code	*Entity Quantity Unit (UCUM)*	Humans	*Person’s height (cm)* *Person’s weight (kg)*
	name	*Entity Name*	EN	Formatted	Text	*Entity Name*	Humans	*Patient name* *Mother name* *Father name* *Neonatal name* *Encounter name*
				Use code	Code	*Entity Name Type Code*	Humans	
	desc	*EntityDesc*	ED	Data	Text or Multimedia	*Entity Describe*	Humans	*Organization Describe*
	existenceTime	*EntityExist Time*	TS	IVL<TS>	Values	*Entity Existence Effective Time*	Humans	*Organization existence effective time*
	telecom	*EntityTelecom*	URL	Address	Text	*Entity Telecom Address*	Humans Organizations Place	*home address* *primary home address* *vacation home address*
				Scheme,CS	Code	*Entity Telecom Means Code*	Humans Organizations	*Fttp* *Http* *tel*
				Use code	Code	*Entity Telecom Address Type Code*	Humans Organizations Place	*Patient telephone number* *Work telephone number* *Guardian’s phone number* *Person’s phone number*
				Useable Period	Values	*Entity Telecom Address Useable Period*	Humans Organizations	*Patient telephone number useable period* *Work telephone number useable period* *Guardian’s phone number useable period*

Note: all attributes of Entity are listed in the table. The *entity class code* and *code* only show the code part of the Table 6

### The web-based system for HCDM

Based on HCDM and NHDD, the web-based system (http://222.249.173.28:38646/STDWEB/) was developed to facilitate centralized management for healthcare metadata. Main functions of the system include: data element management (input, search, browse, edit, etc. for data elements and other metadata items, such as data element concepts, value domains, data sets, etc.), import, export of DEs and data sets (excel, word, pdf, XML formats), and system maintenance. Users can be authorized to browse or edit the content of the system. If a user needs to add a new metadata item, or to update an existing one, he/she should apply for user permission firstly, the added or updated metadata must be inspected and approved by authorized organization before publishing.

The system was constructed basing on a cloud architecture and using Java 2 Platform, Enterprise Edition (J2EE). It supports the access from cross-platform, cross-region and cross-network operations, and also supports the standards of simple object access protocol, eXtensible Markup Language (XML), workflow management coalition, etc. Distributed transaction processing mechanism was adopted to ensure a high consistency of distributed operation transactions and information, to prevent data inconsistency caused by the partial server or network runtime failure of distributed system.

**Fig. 4:**
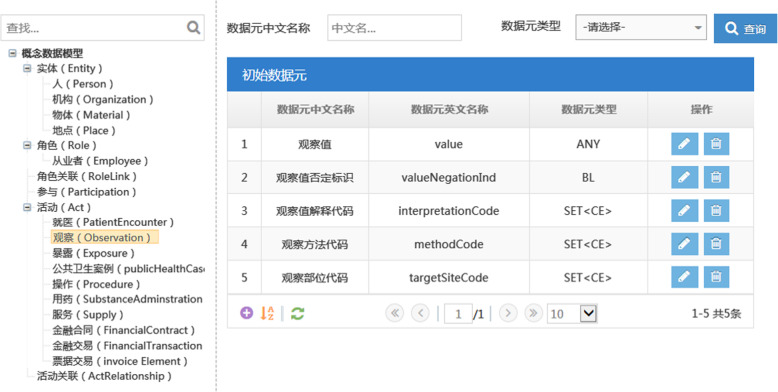
A display interface of initial DE in the system, including initial DE’s Chinese name, English name, data type, edit and delete function

**Fig. 5:**
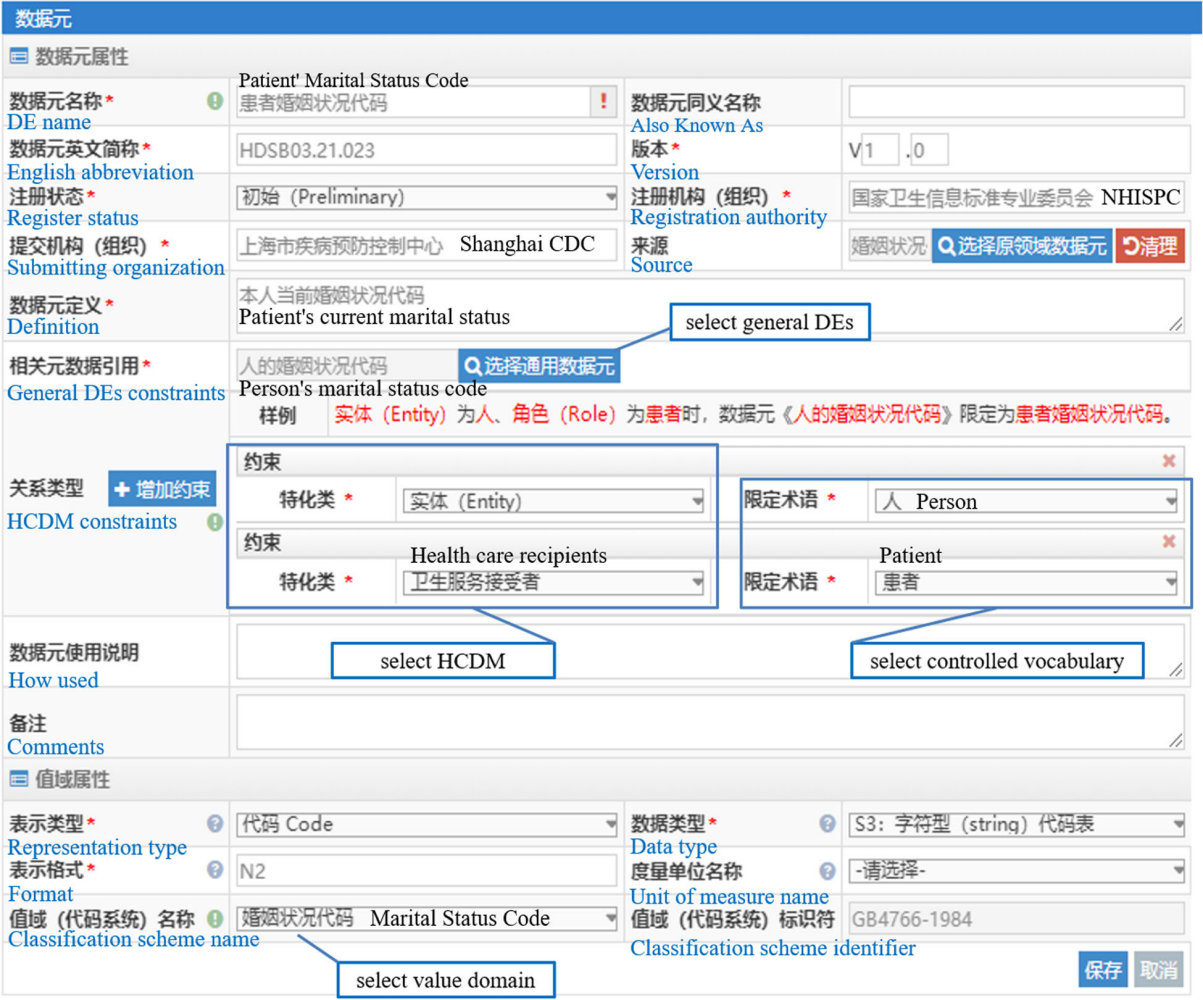
The input and revise interface of domain DE in the system. Domain DEs are standardized through 22 metadata descriptions, including 14 data element attributes and 6 value code attributes. Among them, data element attributes reflect relationships among domain DEs, HCDM and NHDD. Value domain attributes reflect the relationship between domain DEs and controlled vocabularies

The relationships among HCDM, data elements and value domains are connected through web links in the system. The value sets of general DEs are linked to the classification scheme which contains the value codes of general DEs and domain DEs. Figure 4 is a display interface of initial DE in the system, including DE’s Chinese name, English name, data type and edit function. The input and interface of domain DEs are shown in Fig. 5. For instance, by constraining “entity” and “role” (from HCDM) to “person” and “patient” (from controlled vocabularies), general DE “person’s marital status code” will be constrained to the domain DE “patient’s marital status code” accordingly.

## Discussion

Our research is focused on developing the HCDM and NHDD to manage healthcare metadata. There are some advantages in the paper. Firstly, the approach to constrain the metadata has potential to use other projects such as HL7 FHIR, IHE DEX profile to enable semantic interoperability because our domain-specific metadata appears little different from ISO/IEC 11179 metadata registry approach.

Secondly, when other healthcare organizations want to develop their own specific information systems based on system of HCDM and NHDD, general DEs can be specified or localized in the information system for data collection, representation, storage and exchange. Through data element specialization, the definitions for general data elements in the dictionary are constrained consistently to fit specific scenarios by complying with the controlled vocabularies. The dictionary plays a unified reference role for data element specifications of various domains in this process, in which the meaning of data from multiple sources are consistent or at least comparable.

Thirdly, the object classes in the model can be specified step by step following the hierarchy of classes. The volume increase of domain DEs becomes manageable through the constraint of controlled vocabularies, and furthermore, domain DEs have a high degree of semantic consistency by these metadata.

Lastly, HCDM and NHDD can be extended and improved according to future information needs. Compared with HL7 RIM, the HCDM is better suited to practical needs of health data standards management in China. The classes and attributes of HCDM can be appropriately adjusted and extended with the growth or change of health metadata, but the core class will be stable to ensure consistency with related standards. In addition, domain metadata items can be added or revised along with the changes in the health data itself.

The literature [51] achieves syntactic and semantic interoperability between clinical care and research domains by developing a federated semantic metadata registry framework. Although our research is also aimed to develop a metadata framework to enable semantic interoperability, their mechanism is mainly based on the ISO 11179, whereas ours mainly based on HL7 RIM in developing the national HCDM and made a standardized description of metadata according to ISO/IEC 11179.

Some limitations must be acknowledged in the paper. One is that some emerging standards such as HL7 FHIR have not yet been adopted in our development process, and there would be challenges in maintaining consistency with existing standards and achieving interoperability with other international projects in the future. In subsequent work, we will consider those standards such as FHIR and IHE DEX in standard updating according to actual needs. The other is that, despite the availability of the web-based systems, the creation of the standardized domain DEs is relatively complex and we need to strengthen staff training and advancing the implementation process.

## Conclusions

In summary, based on HL7 RIM and actual health services demands, we built the HCDM to provide a unified metadata reference for multi-source data standardization and management, and then developed a web-based system to for its implementation and evaluation. Through a period of practical use, this project has been proved feasible in its designed function.

## Methods

Health data standards were adapted based on the needs of the national health system. 55 data sets (4640 data items) were used as the main data source to establish HCDM, which are currently categorized into 7 health business domains (Table 1). Data sets are related to medical activities enacted by the Chinese National Health Information Standard Committee [52]. We are mainly concerned with the health information of individuals, so data sets of health supervision which are more about information of groups were removed from the data source.

**Fig. 6:**
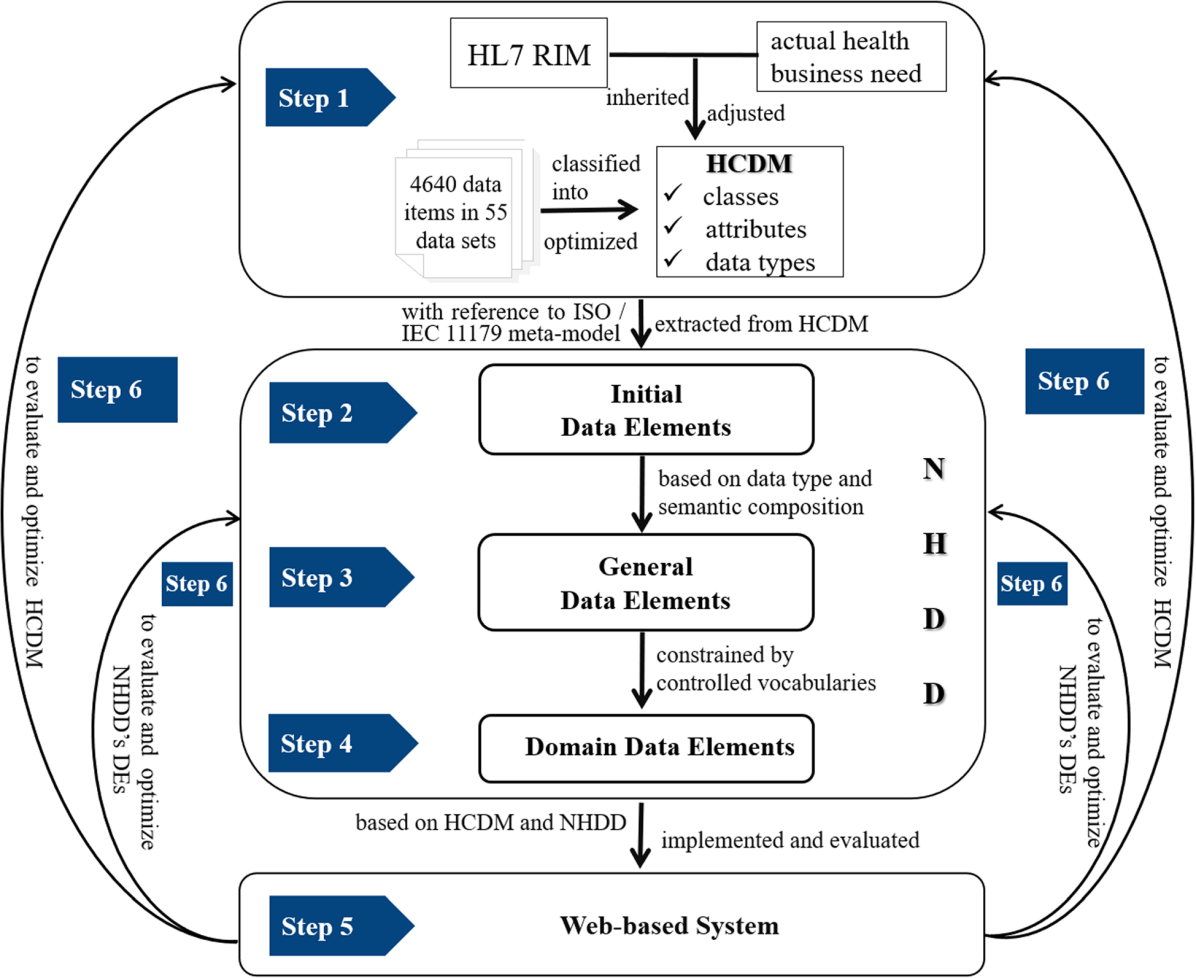
The work process of HCDM, NHDD and its implementation. There are mainly 6 steps for our work process: step 1 establishes the HCDM, step 2 extracts the initial DE, step 3 constructs the general DE, step 4 develops controlled vocabularies and domain Des, step 5 develops the web-based system and step 6 evaluates and optimize HCDM and NHDD

The development process and its implementation of this work mainly included 6 steps as follows (Fig. 6):

Step 1: Establish the HCDM. The HCDM establishment mainly came from HL7 RIM and Chinese actual health information needs, and adjusted and optimized basing on the classification results of data items. Firstly, six classes and their attributes directly used the contents of HL7 RIM’s classes. Secondly 4640 data items from 55 data sets were classified into six classes of HCDM. Subclasses and attributes of HL7 classes were adjusted and trimmed according to actual classification results.

Step 2: Extract the initial DE according to the knowledge on the ontological representation of the ISO/IEC11179 metamodel and the HCDM. The mapping relationships were found between ISO/IEC 11179 metamodel and HCDM to describe data elements.

Step 3: Construct the general DE. The generation of general data elements was constrained by the HCDM and the initial DE. The normalized description of general DEs adopted ISO/IEC 11179 metamodel.

Step 4: Develop controlled vocabularies and domain DEs. Based on standard WS 364 and HL7 vocabularies, controlled vocabularies (value sets) were developed to ensure that all the data items have been included in selected data sets in developing domain DEs. As such, all general DEs and their value sets were standardized to form NHDD.

Step 5: Develop the web-based system. Based on HCDM and NHDD, a web-based system was developed to implement the centralized management for healthcare metadata, and also to evaluate and optimize the HCDM and NHDD. The system is running on the Chinese Health Information Standard Portal and is managed by the national health statistics and information centre.

Step 6: Evaluate and optimize HCDM and NHDD. Based on problems occurred in system’s construction and implementation, the model and DEs in NHDD were further adjusted and optimized to meet actual requirements in health information interoperability.

## Data Availability

The datasets used and/or analysed during the current study are available from the corresponding author on reasonable request.

## Abbreviations

HL7: Health Level Seven
HDF: HL7 Development Framework
HL7 v3: HL7 version 3
DE: Data element
RIM: Reference Information Model
HCDM: Health Concept Data Model
NHDD: National Health Data Dictionary
UCUM: Unified Code for Units of Measure
ISO/IEC: International Organization for Standardization/International Electrotechnical Commission
WS: Wei Sheng (Standard)
IHE DEX: Integrating the Healthcare Enterprise Data Element Exchange
HL7 FHIR: HL7 Fast Health Interoperability Resources
